# AZD6738 reverses the LPS-induced corneal inflammatory transcriptional program through a conserved transcription factor network converging on NF-κB

**DOI:** 10.3389/fmed.2026.2005671

**Published:** 2026-10-26

**Authors:** Qin Ye, Kaiping Chen, Longxiang Huang, Yinqi Chen, Da Chen, Biting Zhou, Yihua Zhu

**Affiliations:** 1 Department of Ophthalmology, The First Affiliated Hospital of Fujian Medical University, Fuzhou, Fujian, China; 2 Department of Ophthalmology, National Regional Medical Center, Binhai Campus of the First Affiliated Hospital, Fujian Medical University, Fuzhou, Fujian, China; 3 Fujian Institute of Ophthalmology, The First Affiliated Hospital of Fujian Medical University, Fuzhou, Fujian, China; 4 Fujian Provincial Clinical Medical Research Center of Eye Diseases and Optometry, The First Affiliated Hospital of Fujian Medical University, Fuzhou, Fujian, China; 5 Department of Ophthalmology, Fujian Medical University Union Hospital, Fuzhou, Fujian, China

**Keywords:** AZD6738, bacterial keratitis, lipopolysaccharide, NF-κB, transcription factor regulon

## Abstract

**Background:**

Vision loss in bacterial keratitis arises primarily from the host inflammatory response rather than the infection itself, and antimicrobial resistance narrows the benefit achievable with antibiotics alone. AZD6738, an inhibitor of ataxia telangiectasia and Rad3-related kinase (ATR), suppresses lipopolysaccharide (LPS)- induced corneal inflammation in mice, but the transcriptional basis of this effect is uncharacterized.

**Methods:**

Mice received intrastromal LPS injection and were assigned to control (CTL), LPS and AZD6738-treated (ATRi) groups (*n* = 3 each). Corneas harvested at 48 h were analyzed by RNA sequencing with DESeq2-based differential expression, KEGG enrichment, regression-based reversal quantification and TRRUST v2 regulon gene set enrichment analysis (GSEA). Cross-species conservation was assessed in a human bacterial keratitis transcriptome (GSE241715; 4 cases, 7 controls). In RAW264.7 macrophages, p65-3 × Flag overexpression with LPS stimulation tested the effect of AZD6738 on NF-κB-driven transcription and Ser536 phosphorylation; tolerability was assessed in HCE-T corneal epithelial cells.

**Results:**

LPS upregulated 1,622 and downregulated 140 corneal genes (padj <0.05, |log_2_FC| > 1), enriched in 80 immune-related pathways. Regression of the treatment effect on the LPS effect indicated reversal of approximately 57% of LPS-induced changes, and 51.2% of LPS-upregulated genes were significantly reversed. Regulon analysis defined an inflammatory transcription factor (TF) program spanning NF-κB, IRF, STAT, AP-1 and myeloid families; cross-species normalized enrichment score (NES) values were rank-correlated (Spearman *ρ* = 0.62, *n* = 214, *P* = 3.4 × 10^−24^). NFKB1, RELA, SPI1 and STAT1 were significant in both species, and AZD6738 shifted their regulon activity to negative values (NES −2.81 to −2.56). In p65-overexpressing RAW264.7 cells, AZD6738 reduced NF-κB-driven transcription and Ser536 phosphorylation.

**Conclusions:**

AZD6738 reverses a substantial part of the LPS-induced corneal inflammatory transcriptome and suppresses an inflammatory TF program whose regulons are conserved in human bacterial keratitis, supporting ATR inhibition as an adjunct to antimicrobial therapy in bacterial keratitis.

## 1 Introduction

Bacterial keratitis (BK) continues to rank among the leading infectious causes of corneal blindness, with the heaviest burden in low- and middle-income countries (1, 2), and corneal opacification from infection and its sequelae accounts for a substantial share of potentially preventable visual loss worldwide (3). Vision loss results mainly from corneal scarring and fibrotic repair rather than from the infection itself, and scarring remains a leading indication for corneal transplantation (4, 5). Antimicrobial resistance among corneal isolates increasingly limits therapy (6), yet topical antibiotics remain the mainstay for eradicating the organism (7), and management still centers on antimicrobial therapy rather than on the host inflammatory response (8).

Lipopolysaccharide (LPS), the principal outer-membrane constituent of Gram-negative bacteria, is engaged by Toll-like receptor 4 (TLR4) and its adaptors to activate NF-κB (9). In the cornea, TLR4-mediated NF-κB signaling in epithelial cells drives LPS-induced inflammation (10), and blocking this axis reduces cytokine production and bacterial load in bacterial keratitis (11). Resident corneal macrophages amplify the response and, through their polarization state, balance pro- and anti-inflammatory programs (12, 13); LPS drives these cells toward an M1 signature (14), whereas promoting M2 polarization resolves corneal inflammation and limits tissue damage (15, 16). Beyond the acute response, innate immune programs persist in late-stage human keratitis (17); which transcriptional regulators sustain this inflammatory program, and whether they can be targeted pharmacologically, remain unclear.

ATR is the central kinase of the replication-stress response and preserves genomic integrity by stabilizing stalled replication forks (18, 19). DNA damage activates NF-κB through nucleocytoplasmic shuttling of NEMO/IKK*γ* (20) and is a direct driver of inflammatory signaling (21), but inflammatory signaling also generates DNA damage, so the two processes are reciprocally linked and the net effect depends on the biological context (22). Accordingly, activity of the DNA damage response can promote rather than restrain inflammation: the ROS–ATM–CHK2 axis drives M1 polarization of macrophages (23), DNA damage response activity increases LPS-stimulated TNF-α production in macrophages (24), and activation of the ATR–CHEK1 axis promotes production of pro-inflammatory mediators in epithelial cells (25). The direction is not fixed, however, because in DNA-damage-driven settings ATR inhibition amplifies radiation-induced inflammatory gene expression (26) and interferon signaling (27). In our previous study (28), LPS triggered ATR phosphorylation in macrophages, and pharmacologic ATR inhibition dampened NF-κB activity, which places ATR upstream of NF-κB in LPS-induced inflammation rather than downstream of DNA damage. Whether ATR supports NF-κB activity through its replication-stress function or through a non-canonical role in inflammatory transcription remains unresolved.

AZD6738 (ceralasertib), an orally available and selective inhibitor of ATR originally developed for oncology (29), and the magnitude of clinical benefit from ceralasertib has been associated with the inflammatory state of the tumor (30), suggesting that ATR inhibition interacts with inflammatory programs in human tissue. In a BALB/c model of LPS-induced keratitis, AZD6738 delivered by subconjunctival injection with topical eye drops attenuated corneal opacity and neovascularization, reduced macrophage infiltration and collagen deposition, and shifted macrophages away from the M1 phenotype via NF-κB (28). The transcriptional basis of this response remains unresolved: the scale of the LPS-induced transcriptome and the signaling pathways it engages, the extent to which a pharmacological intervention reverses these changes, the transcription factors (TFs) that organize the inflammatory program, and the conservation of this axis in human disease have not been characterized. In the present study, we integrated bulk RNA sequencing-based transcriptomic profiling of the LPS-induced mouse corneal inflammation model with cell-level validation to characterize the transcriptional basis of AZD6738-mediated anti-inflammatory effects. We show that AZD6738 reverses approximately 57% of the LPS-induced transcriptomic shift, and that the suppressed program is organized by a conserved inflammatory transcription factor network (NFKB1, RELA, SPI1, STAT1) whose regulon activity is likewise elevated in human bacterial keratitis. In p65-overexpressing macrophages, AZD6738 reduces NF-κB-driven transcription and p65 Ser536 phosphorylation. Together, these results place ATR upstream of the NF-κB-dependent inflammatory program in the cornea and support its inhibition as a host-directed adjunct to antimicrobial therapy in bacterial keratitis.

## 2 Materials and methods

### 2.1 Animals

Male BALB/c mice aged six to eight weeks were obtained from Skbex Biotechnology (Henan, China) and kept under standard conditions: temperature 23–25 °C, relative humidity 50% ± 10%, and a 12 h light–12 h dark cycle, with food and water provided *ad libitum*. The experimental protocol was reviewed and approved by the Laboratory Animal Welfare and Ethics Committee of Fujian Medical University (IACUC FJMU 2024-Y-0915), and all procedures followed institutional guidelines for animal care.

### 2.2 LPS keratitis model and AZD6738 treatment

Anesthesia consisted of 2% isoflurane inhalation followed by topical 0.5% tetracaine (Zhongshan Ophthalmic Center, Guangzhou, China) applied to both eyes. Lipopolysaccharide (LPS; from Escherichia coli O55:B5, HY-D1056, MedChemExpress, USA) was reconstituted in sterile normal saline at 1 mg/mL, and keratitis was induced by intrastromal injection of 2 μL of this solution into the central cornea of both eyes with a 33G Hamilton microsyringe (Model 701 RN SYR; Hamilton Co., USA); one experienced ophthalmologist performed all injections. AZD6738 (ceralasertib, HY-19323, MedChemExpress, USA), prepared from a dimethyl sulfoxide (DMSO) stock and diluted in normal saline to 100 μM, was given immediately after modeling as a 10 μL subconjunctival injection followed by topical drops four times daily (qid) for 48 h. Vehicle controls received normal saline with 0.1% DMSO subconjunctivally and topically on the same schedule. The transcriptomic cohort comprised three groups (*n* = 3 each): CTL (bilateral intrastromal PBS plus vehicle), LPS (bilateral intrastromal LPS plus vehicle) and ATRi (bilateral intrastromal LPS plus AZD6738). At 48 h after modeling the mice were euthanized by cervical dislocation; both eyes were enucleated at once, the corneas excised and pooled per animal as one biological replicate, snap-frozen in liquid nitrogen and kept at −80 °C until RNA extraction. Animals were monitored daily throughout the experiment in accordance with institutional guidelines. Humane endpoints were predefined as body-weight loss of 20% to 25% or corneal perforation. All animals completed the full observation period, and no unplanned endpoints occurred.

### 2.3 Cell culture

HCE-T human corneal epithelial cells (Zhejiang Meisen Cell Technology Co., Ltd., China) were maintained in HCE-T complete medium (CTCC-002-039; Meisen, China), and RAW264.7 murine macrophages (Shanghai Saibaikang Biotechnology Co., Ltd., China) were maintained in RAW264.7-specific medium (iCell-m047-001b; Saibaikang, China). Both cell lines were obtained from commercial cell banks in which they had been authenticated by short tandem repeat (STR) profiling and confirmed to be mycoplasma-free by the supplier. Both lines were grown at 37 °C under 5% CO_2_ in a humidified incubator and fed with fresh medium every day; RAW264.7 cells between passages 3 and 6 were used for experiments.

### 2.4 CCK-8 cell viability assay

For viability testing, HCE-T cells (100 μL per well, 50% density) were grown in 96-well plates to 70% confluence and then exposed for 24 h to medium containing AZD6738 at 0.5, 1, 2.5 or 5 μM. CCK-8 reagent (CK04; Dojindo Laboratories, Japan, 10 μL per well) was then added; after a 1 h incubation, the optical density at 450 nm was recorded on a microplate reader.

### 2.5 F-actin fluorescent staining

Following drug exposure, the cells were fixed in 4% paraformaldehyde (15 min, room temperature), permeabilized three times with PBS containing 1% Triton X-100 (5 min each), stained with rhodamine phalloidin working solution (HY-K0903; MedChemExpress, USA) for 30–60 min at room temperature protected from light, and counterstained with DAPI to label nuclei. F-actin morphology was imaged on a PerkinElmer Operetta high-content imaging system, and fluorescence intensity was analyzed in ImageJ.

### 2.6 Scratch assay

Cells were plated in 24-well plates at 90% density and grown overnight to near-confluence (∼100%). With pre-drawn reference lines marking the central area of each well, a straight scratch was made using a 200 μL pipette tip. After two to three gentle PBS rinses to remove loose cells, fresh drug-containing medium was applied. Phase-contrast images taken at 0, 24 and 48 h were analyzed in Fiji, and the wound healing rate was defined as (A_0_h − A_48h_)/A_0h_, where A_0h_ and A_48h_ are the scratch areas at 0 h and 48 h, respectively.

### 2.7 Plasmid construction and transfection

A p65-3 × Flag overexpression plasmid (Fuzhou Zolgene Biotechnology Co., Ltd., China) was introduced into RAW264.7 cells with Lipofectamine 3,000 (L3000015; Thermo Fisher Scientific, USA). Briefly, logarithmic-phase cells were plated in 6-well plates one day before transfection so that they reached 70%–90% confluence on the day of use. For each well, 2.5 μg of plasmid DNA was combined with 5 μL of P3000 reagent in 125 μL of Opti-MEM, while 3.75 μL of Lipofectamine 3,000 was diluted in a separate 125 μL of Opti-MEM; the two solutions were mixed by gentle pipetting and left for 15 min at room temperature. After the growth medium had been aspirated, 250 μL of the mixture was applied and diluted to 1 mL with complete medium, which was replaced with fresh medium 6–8 h later; drug treatment started 48 h after transfection. Four groups were studied: Vector (empty plasmid), Vector + LPS, p65 overexpression (NF-κB-OE) + LPS, and NF-κB-OE + LPS + AZD6738, with LPS (MedChemExpress, USA) at 1 μg/mL and AZD6738 at 2.5 μM for 24 h.

### 2.8 Quantitative real-time PCR (qRT-PCR)

RNA was isolated from the cells of each group using TRIzol reagent (Invitrogen, USA), and RNA quantity and purity (A260/A280) were checked spectrophotometrically. cDNA was synthesized with the RevertAid First Strand cDNA Synthesis Kit (Thermo Fisher Scientific, USA). Quantitative PCR used SYBR Green Real-Time PCR Master Mix (Toyobo, Japan) on a Rotor-Gene Q system (QIAGEN, Germany); every reaction was run in triplicate. With Gapdh as the reference gene, *Il6*, *Tnf* and *Il1b* mRNA levels were derived by the 2^−*ΔΔ*Ct^ method, and primer sequences appear in Supplementary Table S1.

### 2.9 Western blot

Cells were lysed in RIPA buffer (CWBio, China) containing PMSF and phosphatase inhibitors for 30 min on ice; after centrifugation at 4 °C to clear debris, protein content was measured with a BCA kit. Equal amounts of protein (30–60 μg) were resolved by SDS-PAGE and electroblotted onto PVDF membranes (Millipore, USA), which were blocked in 5% skim milk in 1× TBS-T for 1 h at room temperature and probed overnight at 4 °C with antibodies recognizing p-p65 (Ser536) (80379-2-RR; Proteintech, China), p65 (80979-1-RR; Proteintech, China), TNF-α (80258-6-RR; Proteintech, China) and GAPDH (AF5718; R&D Systems, USA). After TBS-T washes, the blots were incubated with the corresponding secondary antibodies (1:10,000; ZSGB-BIO, China) for 1 h at room temperature, developed by enhanced chemiluminescence, and quantified by densitometry in ImageJ.

### 2.10 Transcriptome sequencing

Library preparation and sequencing were carried out by Biomarker Technologies Co., Ltd. (China). Corneal RNA was isolated with TRIzol (Life Technologies, USA); concentration and purity were measured on a NanoDrop 2,000, exact amounts were determined with Qubit 2.0, and integrity was checked on an Agilent Bioanalyzer 2,100 (RNA Nano 6,000 Assay Kit). One microgram of qualified RNA per sample served as input for libraries built with the NEBNext Ultra RNA Library Prep Kit for Illumina (NEB, USA): mRNA bearing oligo(dT) beads was enriched and fragmented under heat in the presence of divalent cations; first-strand cDNA was primed with random hexamers, and second-strand synthesis used DNA Polymerase I with RNase H; after cleanup and size selection on AMPure XP beads (Beckman Coulter, USA), adapters were removed with USER enzyme ahead of PCR and the products re-purified. Libraries that passed QC on the Bioanalyzer 2,100 and Q-PCR were sequenced in paired-end 150 bp mode on an Illumina platform.

Each sample yielded no less than 5.41 Gb of clean data, with Q30 base percentages of at least 92.20% and per-sample mapping rates ranging from 89.04% to 95.72%.

### 2.11 Bioinformatic analysis

#### 2.11.1 Quality control and alignment

Low-quality reads, adapter remnants and reads containing N bases were trimmed to yield clean reads, from which Q20, Q30, GC content and duplication metrics were computed. The clean reads were mapped to Mus_musculus. GRCm38 (Ensembl release 95) with HISAT2 (per-sample mapping rates 89.04%–95.72%), transcripts were assembled and FPKM values quantified with StringTie, and a gene-level count table was produced for differential testing.

#### 2.11.2 Differential expression analysis

Differential expression between groups was tested with the DESeq2 R package, which models counts with the negative binomial distribution (design ∼group); *P* values came from the Wald test as implemented in DESeq2 and were adjusted by the Benjamini–Hochberg procedure. A gene was called differentially expressed only when its adjusted *P* value (padj) was below 0.05 and its absolute log_2_ fold change exceeded 1, evaluated for LPS vs. CTL, ATRi vs. LPS and ATRi vs. CTL; the direction of change followed the ordering of each comparison.

#### 2.11.3 Principal component analysis

For a sample-level overview, an expression matrix transformed with the DESeq2 variance-stabilizing transformation (vst) was summarized by principal component analysis (prcomp), and the first two components were plotted to show group separation.

#### 2.11.4 KEGG analysis

With all expressed genes as background, gene symbols were converted to ENTREZ IDs with bitr (clusterProfiler R package), and KEGG over-representation was tested with enrichKEGG under Benjamini–Hochberg correction, taking padj <0.05 as the significance threshold. Each of the four differential gene sets (LPS-upregulated, LPS-downregulated, treatment-upregulated, treatment-downregulated) was analyzed in turn, and pathways significant in both the LPS-upregulated and treatment-downregulated sets were treated as shared.

#### 2.11.5 Gene set enrichment analysis (GSEA)

Gene set enrichment analysis (GSEA; clusterProfiler) was run on pathway and TF-regulon (TF-target) sets restricted to 5–2,000 genes. Mouse genes were pre-ranked by the DESeq2 statistic and human genes by the limma moderated t; the permutation seed was fixed at 123 for reproducibility. Benjamini–Hochberg-adjusted padj <0.05 defined significance.

#### 2.11.6 Reversal analysis

The reversal of LPS-induced transcriptional changes by AZD6738 was quantified at three levels. (1) Global level: linear regression was fitted with the LPS effect (log_2_FC of LPS vs. CTL) as the independent variable and the treatment effect (log_2_FC of ATRi vs. LPS) as the dependent variable, and the mean reversal proportion was estimated as the absolute value of the slope. (2) Gene level: LPS-significant differentially expressed genes were classified into fully reversed, partially reversed, and unreversed categories according to the direction and significance of the two effects; reversal proportions were summarized for each direction, and KEGG analysis was performed on reversed genes. (3) Pathway level: among the 74 KEGG pathways significantly enriched in both the LPS-upregulated and treatment-downregulated sets, the nine pathways most directly related to innate immunity and inflammation were selected; for each of them, linear regression of the treatment effect on the LPS effect was fitted gene by gene, and the pathway reversal rate was defined as the negative of the slope.

#### 2.11.7 Transcription factor (TF) analysis

Target over-representation analysis of the differentially expressed gene sets (enricher, padj < 0.05) was performed based on the TRRUST v2 database (mouse) (31), and GSEA was run on all TF regulons (parameters as above). For the cross-species conservation analysis, all human TRRUST v2 regulons (238 TFs) were mapped to mouse genes via orthologous gene symbol mapping; the same regulon definition was applied to both species, GSEA was performed separately in each, and the consistency of NES between the two species was assessed by Spearman rank correlation.

#### 2.11.8 Validation in a human keratitis dataset

The public dataset GSE241715 (FFPE MACE 3′ RNA-seq) contains infectious keratitis and healthy control samples. Because the mouse model was LPS-induced, the analysis was restricted to the bacterial keratitis subgroup: after excluding fungal and viral samples, 4 bacterial keratitis samples and 7 healthy controls were retained. The expression matrix was filtered by row (rowMeans ≥ 0.5) and transformed as log_2_(counts + 1), and differential expression between bacterial and control samples was analyzed with limma (lmFit + eBayes), with padj <0.05 and |log_2_FC| > 1 defining differentially expressed genes. Mouse signature gene sets defined by the differential analysis (LPS-upregulated, LPS-downregulated, AZD6738-reversed, and treatment-upregulated) were mapped to the human data for GSEA, and differences in signature activity between healthy controls and disease samples were assessed by an exact permutation test (difference in group means, full enumeration).

### 2.12 Statistical analysis

Bioinformatic analyses were performed in R (version 4.6.1). Data from the wet-laboratory experiments were analyzed with SPSS 29 (IBM, USA); comparisons among multiple groups were performed by one-way ANOVA, with Games-Howell *post hoc* tests applied when variances were unequal. Differences in human signature activity between groups were assessed by exact permutation tests. Data are presented as mean ± SD with *n* ≥ 3. *P* < 0.05 was considered statistically significant.

## 3 Results

### 3.1 LPS induces transcriptomic changes in the mouse cornea

Corneal transcriptomes were sequenced for nine BALB/c mice [CTL, LPS, and LPS plus AZD6738 (ATRi); *n* = 3 per group]. Principal component analysis (PCA) separated the LPS group from CTL along the first principal component, with the ATRi group positioned between them, consistent with partial restoration of the transcriptional state toward the control profile after AZD6738 treatment (Figure 1A).

**Figure 1:**
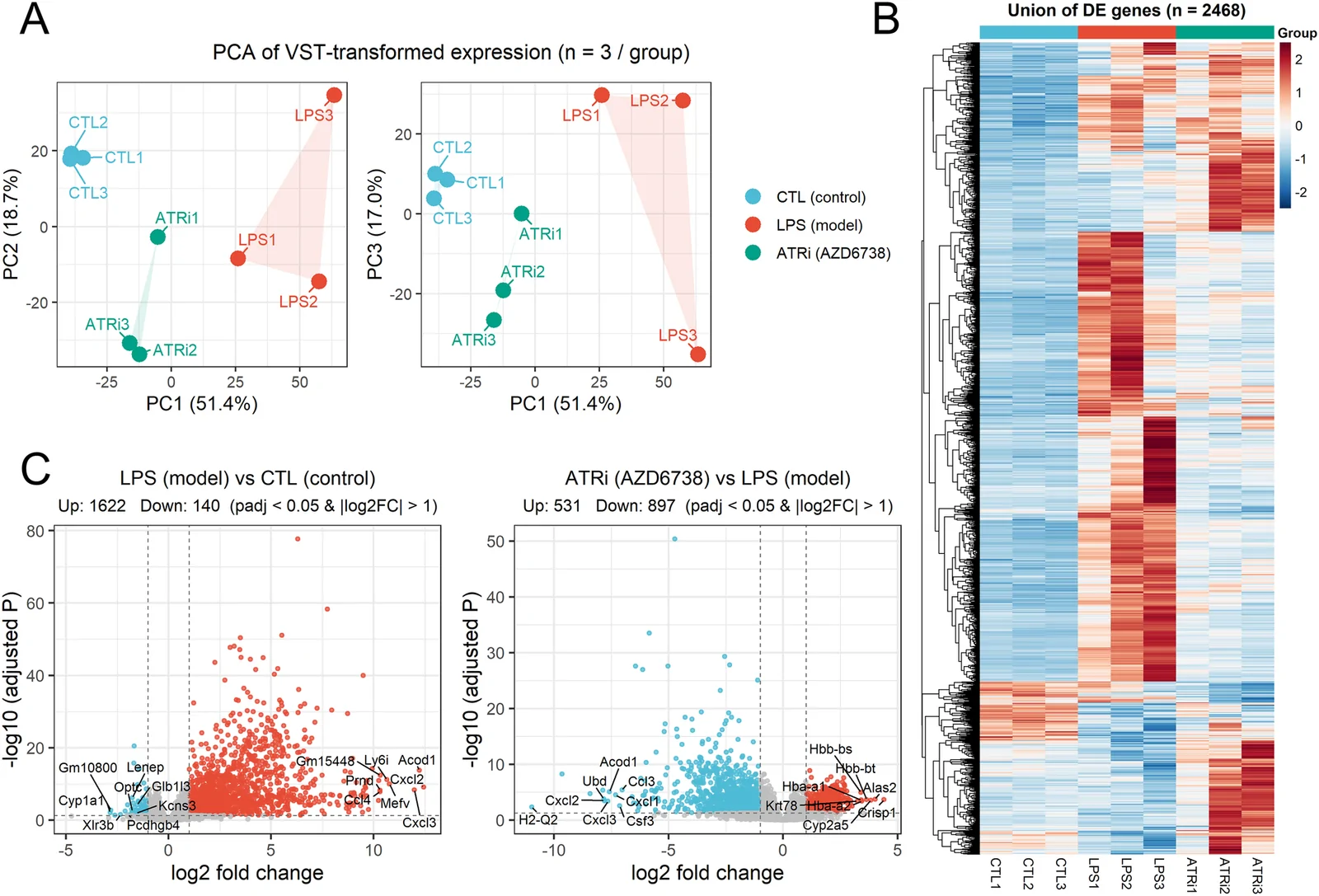
LPS-induced transcriptomic changes and effects of AZD6738 treatment in the mouse cornea. **(A)** Principal components of the three groups; **(B)** heatmap of row z-scores across the 2,468-gene union; **(C)** volcano plots for LPS vs CTL and ATRi vs LPS.

Differential expression analysis of the three pairwise comparisons (DESeq2) yielded a union of 2,468 significant differentially expressed genes. In the heatmap, these genes deviated from the control expression state in the LPS group and shifted back toward it after AZD6738 treatment, in line with the between-group relationships observed by PCA (Figure 1B).

The LPS vs. CTL comparison yielded 1,622 upregulated and 140 downregulated genes, an asymmetric distribution dominated by upregulation; the ATRi vs. LPS comparison yielded 897 downregulated and 531 upregulated genes (Figure 1C). The LPS-induced upregulated genes mainly comprised inflammatory and coagulation-related genes such as *F10*, *Slpi*, *Cxcl10*, *Itgb2* and *Ptprc*, and the genes downregulated by AZD6738 overlapped substantially with this set. Complete lists of differentially expressed genes for the three comparisons, and their union, are provided in Supplementary Table S2.

### 3.2 Differentially expressed genes are enriched in immune and inflammatory signaling pathways

KEGG enrichment analysis showed that the LPS-upregulated genes were significantly enriched in immune-related pathways, including cytokine–cytokine receptor interaction, TNF, NF-κB, IL-17, NOD-like receptor, chemokine signaling and JAK-STAT signaling, with 80 pathways reaching padj < 0.05. The genes downregulated by AZD6738 treatment were enriched in a set of pathways that largely overlapped with the LPS-upregulated set (74 shared pathways), with the direction of enrichment reversed (Figure 2A). KEGG results for all four differential gene sets are shown in Supplementary Figure S1.

**Figure 2:**
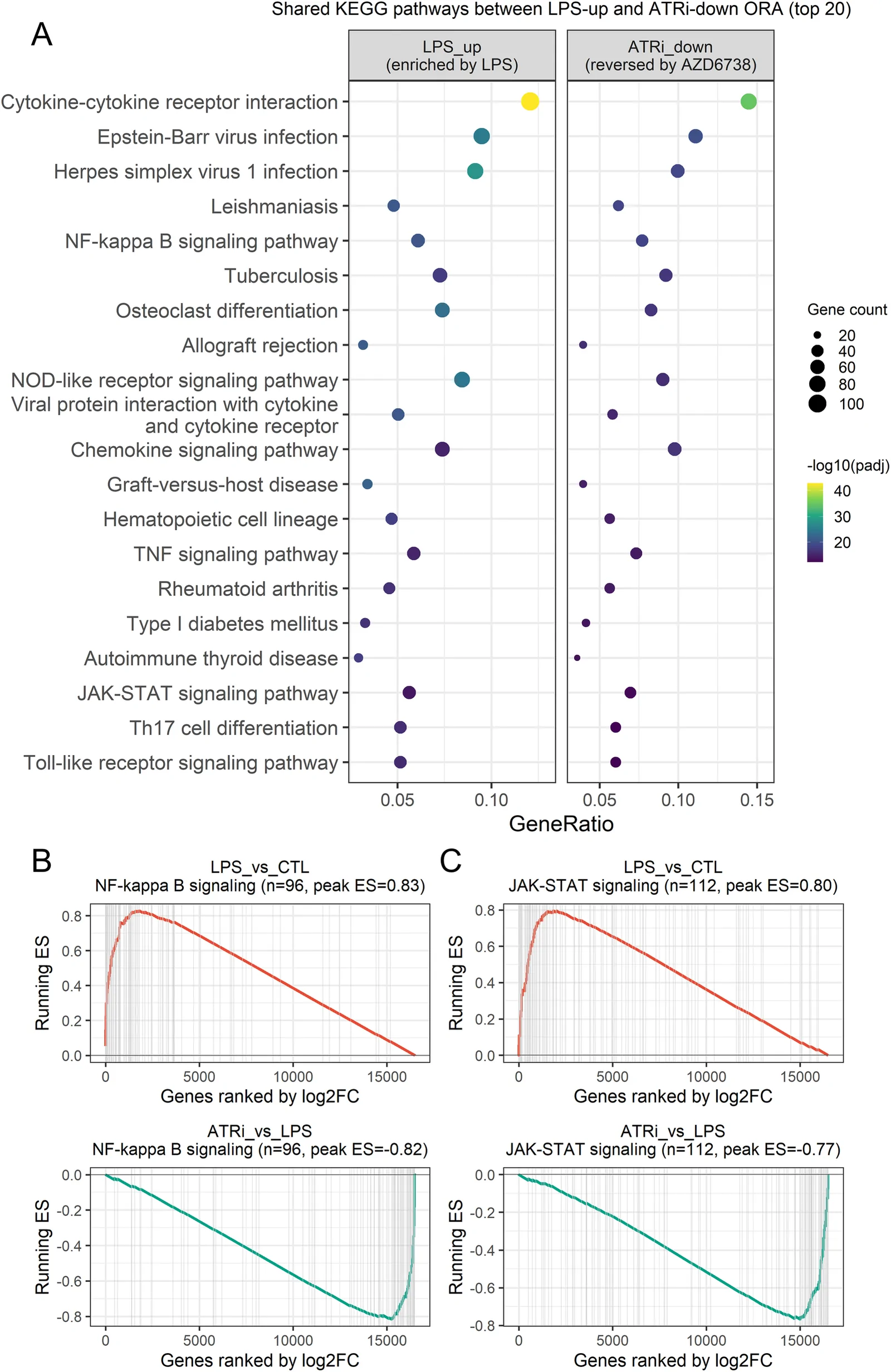
Enrichment of differentially expressed genes in immune and inflammatory signaling pathways. **(A)** Bubble plot of KEGG pathways significantly enriched in both LPS-upregulated and treatment-downregulated gene sets [top 20 of the 74 shared pathways shown; bubble size = number of genes, color = −log₁₀(padj)]; **(B,C)** GSEA enrichment curves for the NF-κB **(B)** and JAK-STAT **(C)** pathways in LPS vs CTL (upper) and ATRi vs LPS (lower).

Analysis of the two core pathways showed significant positive enrichment of both NF-κB and JAK-STAT in LPS vs. CTL and significant negative enrichment of similar magnitude in ATRi vs. LPS, suggesting that both pathways were activated by LPS stimulation and suppressed after AZD6738 treatment (Figure 2B,C).

Grouped expression heatmaps of NF-κB pathway genes (*n* = 96) and JAK-STAT pathway genes (*n* = 112) showed that most genes were upregulated in the LPS group and decreased after AZD6738 treatment (Supplementary Figure S2).

### 3.3 AZD6738 reverses LPS-induced transcriptional changes

The extent of reversal was quantified at the global, gene and pathway levels. At the global level, linear regression of the treatment effect (log_2_FC of ATRi vs. LPS) on the LPS effect (log_2_FC of LPS vs. CTL) was significant (slope = −0.568, 95% CI −0.585 to −0.551), corresponding to a mean reversal of approximately 57% of the LPS-induced expression changes (Figure 3A).

**Figure 3:**
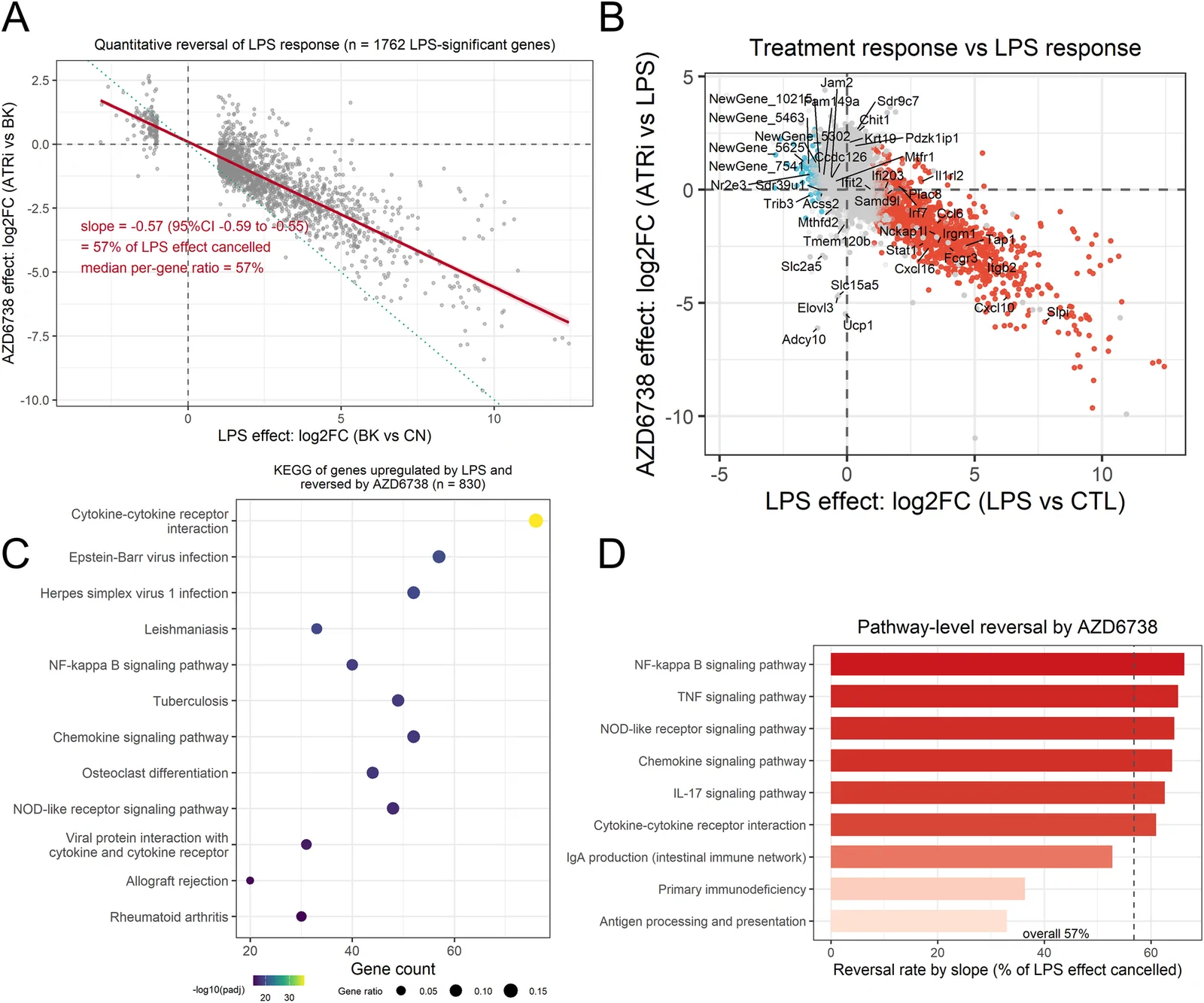
AZD6738 reverses LPS-induced transcriptional changes. **(A)** Linear regression of the treatment effect on the LPS effect (*n* = 1,762; slope = −0.568, corresponding to a mean reversal of approximately 57%); **(B)** Genes were classified using thresholds of padj < 0.05 and |log2FC| > 1 in both contrasts, and fully reversed genes additionally required padj (ATRi vs CTL) ≥ 0.05. Among the 1,622 LPS-upregulated genes, 547 were fully reversed (R2), 283 were partially reversed (R1), 11 were exacerbated (R0), and 781 were not significantly altered in ATRi vs LPS. Among the 140 LPS-downregulated genes, 32 were fully reversed and 1 was partially reversed; **(C)** KEGG analysis of the reversed genes [*n* = 830; 80 significantly enriched pathways, top 12 shown; bubble size = GeneRatio, color = −log₁₀(padj)]; **(D)** reversal rates of the nine immune-related pathways (dashed line = overall reversal rate of 57% for all LPS-significant genes).

At the gene level, LPS-significant genes were classified into reversal categories by the direction and significance of the two effects (Figure 3B): 830 of the 1,622 LPS-upregulated genes (51.2%) were significantly reversed after treatment, as were 33 of the 140 LPS-downregulated genes (23.6%). KEGG analysis of these 830 reversed genes yielded 80 significantly enriched pathways (Figure 3C).

At the pathway level, 74 KEGG pathways were significantly enriched in both the LPS-upregulated and treatment-downregulated gene sets (Figure 2A). Linear regression was fitted for nine KEGG pathways directly related to innate immunity and inflammation, and the negated slope was defined as the pathway reversal rate (Figure 3D). The core pro-inflammatory pathways (NF-κB, TNF, NOD-like receptor, chemokine signaling, IL-17 and cytokine–receptor interaction) showed reversal rates of 61%–66%, above the overall level (dashed line).

### 3.4 Cross-species conservation of core inflammatory transcription factors and their suppression by AZD6738

Target over-representation analysis (ORA) based on the mouse TRRUST v2 database showed that the LPS-upregulated genes were significantly enriched in the target sets of 16 TFs, centered on *Nfkb1*, *Spi1*, *Irf1*, *Rela*, *Stat1*, *Ikbkb* and *Jun*, with *Nfkb1* showing the strongest enrichment. The genes downregulated by AZD6738 were significantly enriched in the target sets of 20 TFs, and the most significantly enriched TFs were consistent with the LPS-upregulated ORA results, suggesting that the transcriptional program suppressed by AZD6738 is driven largely by these inflammatory TFs. LPS-downregulated genes were enriched only in target sets of myogenic differentiation-related TFs (*Myod1* and *Myog*), and no significantly enriched TFs were detected for the treatment-upregulated genes. Regulon GSEA gave results consistent with the ORA (Figure 4A): core inflammatory TFs such as *Rela*, *Nfkb1*, *Irf1*, *Stat1* and *Jun* were significantly activated in LPS vs. CTL and shifted to significantly negative values in ATRi vs. LPS. These TFs span the NF-κB, IRF, STAT, AP-1 and myeloid families and together constitute the core transcriptional regulator*y* axis of LPS-induced corneal inflammation.

**Figure 4:**
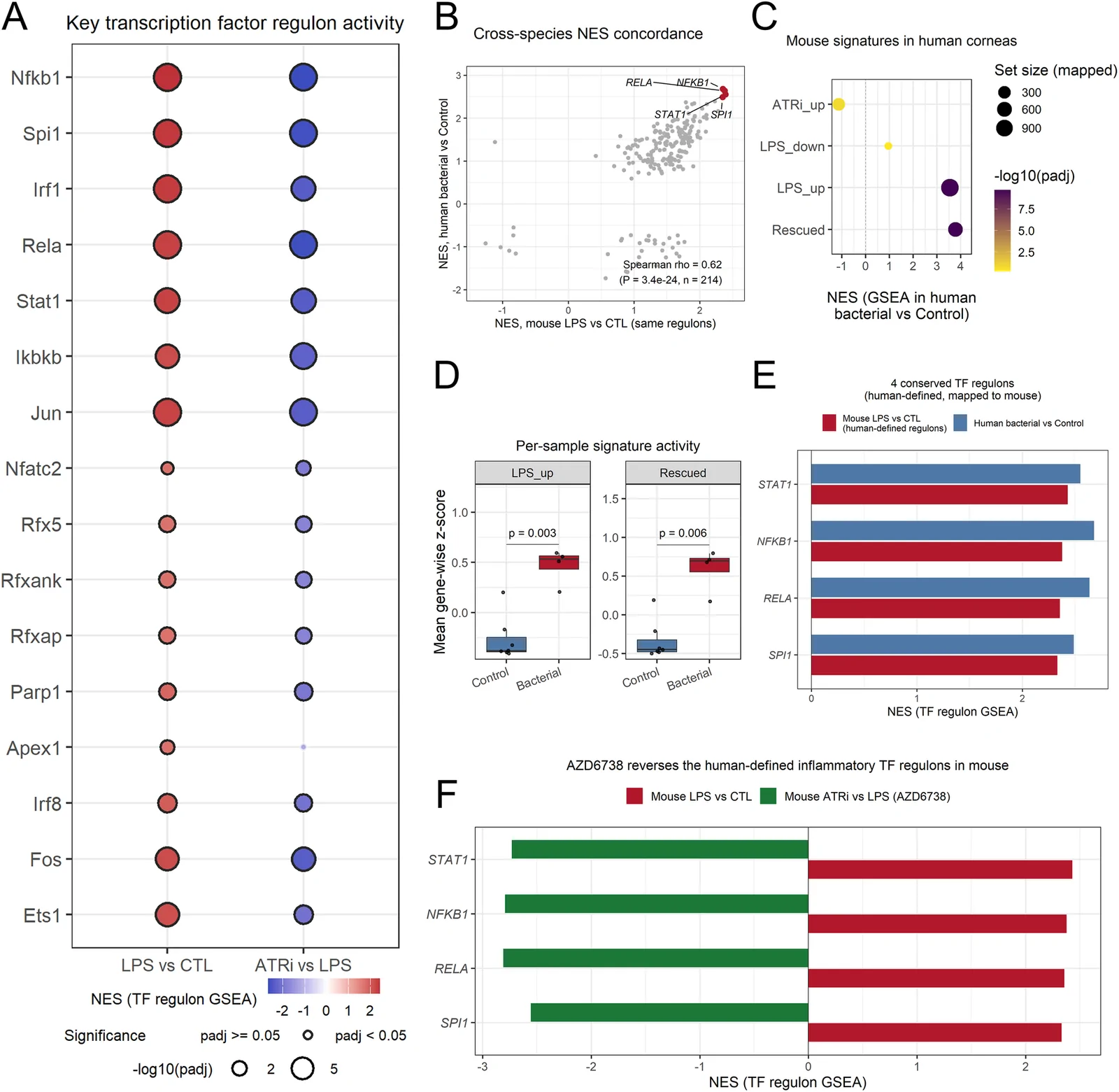
Cross-species conservation of core inflammatory transcription factors and their suppression by AZD6738. **(A)** Dot plot of regulon GSEA for the 16 ORA-significant TFs [dot size = −log_10_(padj), color = NES; outlined dots = padj <0.05]; **(B)** scatter plot of cross-species NES rank correlation (both species analyzed with the same set of human TRRUST v2 regulons; *n* = 214, Spearman *ρ* = 0.62, *P* = 3.4 × 10^−24^; red dots = the four TFs shared between the two species' ORA); **(C)** bubble plot of GSEA for mouse signature gene sets mapped to the human dataset; **(D)** Per-sample signature activity in the human dataset (Control vs Bacterial), where *P* values were derived from an exact permutation test on group labels (4 bacterial vs 7 control samples), in which all 330 possible label assignments were enumerated without random sampling, with two-sided testing; **(E)** NES comparison of the four ORA-shared TFs across species; **(F)** reversal of the four shared TF regulons by AZD6738 (NES all shifted to significant negative values after treatment, padj <0.05).

To test the cross-species conservation of this regulator*y* axis, we used the human corneal MACE 3′ RNA-seq dataset GSE241715. Because the mouse model was LPS-induced, the analysis was restricted to bacterial keratitis; after excluding fungal and viral samples, four bacterial keratitis samples and seven healthy controls were retained, and 1,487 upregulated and 1,381 downregulated genes were identified. Parallel GSEA was performed in both species using all human TRRUST v2 regulons (238 TFs), with orthologous gene symbol mapping used to match mouse genes to their human counterparts. Among the 214 TFs tested in both species, the human and mouse NES values showed a significant positive rank correlation (Spearman *ρ* = 0.62, *P* = 3.4 × 10^−24^; Figure 4B), suggesting overall concordance in transcriptional regulation between the LPS mouse model and human bacterial keratitis.

At the signature level, four signatures defined from the mouse differential analysis (LPS-upregulated, LPS-downregulated, AZD6738-reversed LPS-upregulated, and treatment-upregulated) were mapped to the human dataset for GSEA. The AZD6738-reversed signature showed the highest NES, followed by the LPS-upregulated signature (Figure 4C), and the per-sample activities of both differed significantly between healthy controls and disease samples (Figure 4D), suggesting that the inflammatory program reversed by AZD6738 in the mouse is also significantly activated in human bacterial keratitis.

At the TF level, ORA of the human upregulated genes using human TRRUST v2 identified five significant TFs (*NFKB1*, *RELA*, *SPI1*, *STAT1* and *REL*). Intersecting these with the 16 mouse-significant TFs using orthologous gene symbol mapping retained four cross-species conserved TFs (*NFKB1*, *RELA*, *SPI1* and *STAT1*). GSEA was then run in both species with the human TRRUST v2-defined regulons of these four TFs, and all four regulons were significantly enriched in both species-level comparisons (human bacterial keratitis vs. controls: NES 2.49–2.68, padj ≤ 5.8 × 10^−7^; mouse LPS vs. CTL: NES 2.33–2.43, padj ≤ 1.6 × 10^−10^; Figure 4E). With the same set of human regulons mapped to mouse, AZD6738 treatment significantly suppressed the regulon activity of all four conserved TFs, shifting NES to negative values (−2.81 to −2.56, padj <0.05; Figure 4F).

### 3.5 AZD6738 reduces NF-κB-driven transcription in p65-overexpressing macrophages

To verify at the cellular level the inhibition of the NF-κB axis by AZD6738 predicted by the transcriptomic and regulon analyses, a p65-3 × Flag overexpression plasmid was constructed (Figure 5A) and transfected into RAW264.7 macrophages. Western blotting confirmed that p65 protein levels were significantly elevated after transfection compared with the empty-vector control (Figure 5B). Under LPS stimulation, p65 overexpression further upregulated the mRNA levels of *Il6*, *Tnf* and *Il1b*. Co-treatment with AZD6738 significantly reduced all three (Figures 5C–E), indicating that the NF-κB-driven transcriptional activation is sensitive to AZD6738. At the signaling level, p65 overexpression significantly increased Ser536 phosphorylation, whereas AZD6738 treatment significantly reduced it (Figures 5F–I); the increase in the downstream effector TNF-α was similarly suppressed by AZD6738 (Figure 5J). Together, these results suggest that ATR inhibition suppresses the transcriptional activity of NF-κB p65 by blocking its Ser536 phosphorylation, thereby reducing downstream pro-inflammatory cytokine expression. Because no parallel viability measurement was performed, selective depletion of cytokine-active macrophages by AZD6738 cannot be excluded as a contributor to these reductions (Figures 5C–E).

**Figure 5:**
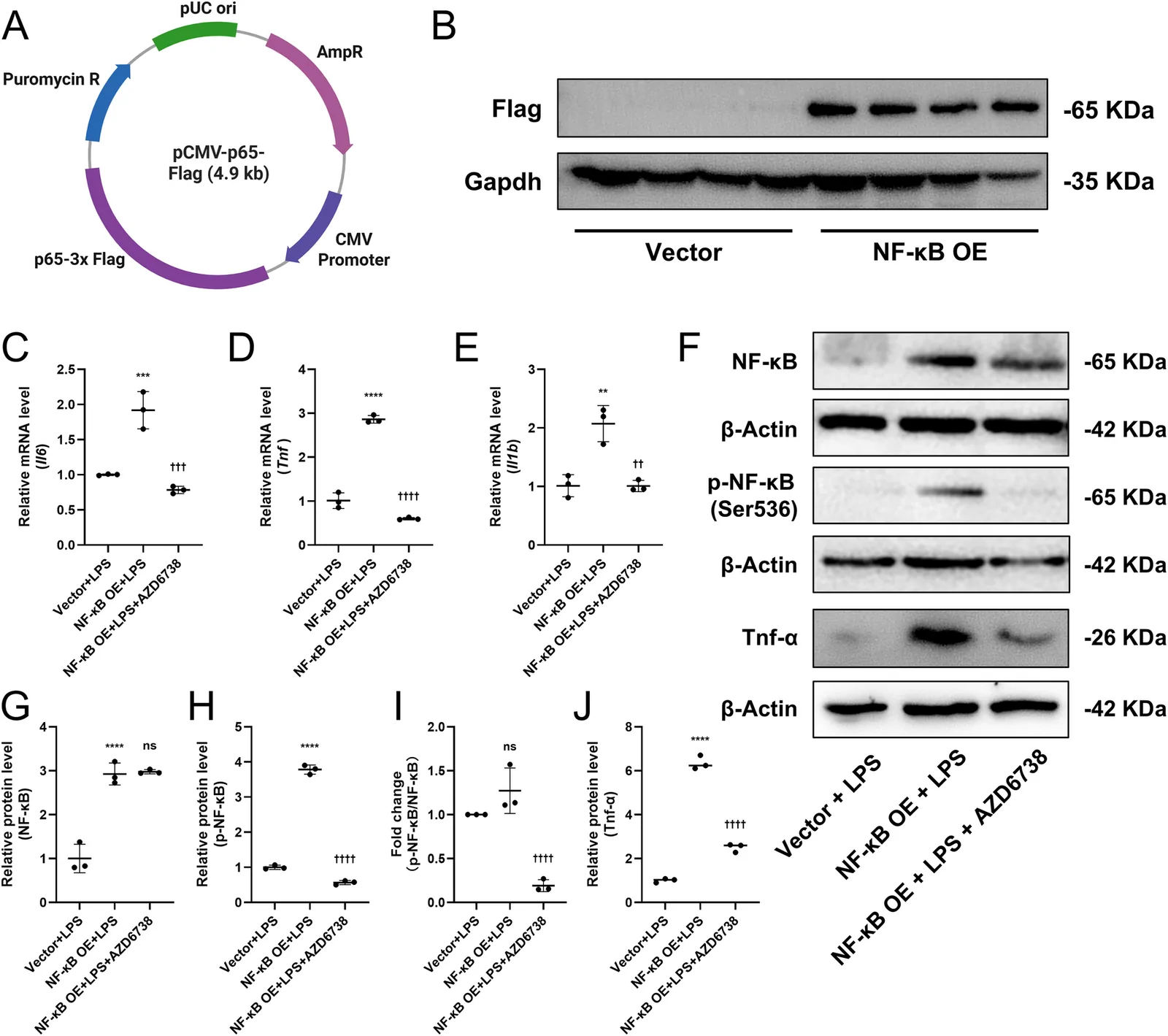
AZD6738 suppresses the inflammatory transcriptional program by reducing NF-κB Ser536 phosphorylation. **(A)** Schematic of p65-3 × Flag overexpression plasmid construction. **(B)** Western blot verification of p65 overexpression efficiency. **(C–E)** qRT-PCR detection of *Il6* **(C)**, *Tnf* **(D)**, and *Il1b* **(E)** mRNA expression levels under p65 overexpression conditions. **(F)** Western blot detection of p65, p-p65 (Ser536), and TNF-α protein expression. **(G–J)** Densitometric quantification of p65 **(G)**, p-p65 **(H)**, p-p65/p65 ratio **(I)**, and TNF-α **(J)** protein levels. Data are presented as mean ± SD (*n* = 3). **P < 0.01, ***P < 0.001, ****P < 0.0001 vs. Vector + LPS; ††*P* < 0.01, †††*P* < 0.001, ††††*P* < 0.0001 vs. NF-κB-OE + LPS group.

### 3.6 AZD6738 does not impair corneal epithelial cell viability, cytoskeleton, or migration at tested concentrations

To ask whether AZD6738 is tolerated at the ocular surface, HCE-T cells were treated with AZD6738 (0.5, 1, 2.5, or 5 μM) for 24 h. Relative viability did not differ significantly among the four AZD6738 concentrations and the vehicle (Figure 6A; *n* = 3, *P* > 0.05); the concentration used in the macrophage experiments described in section 3.5 (2.5 μM) falls within this range.

**Figure 6:**
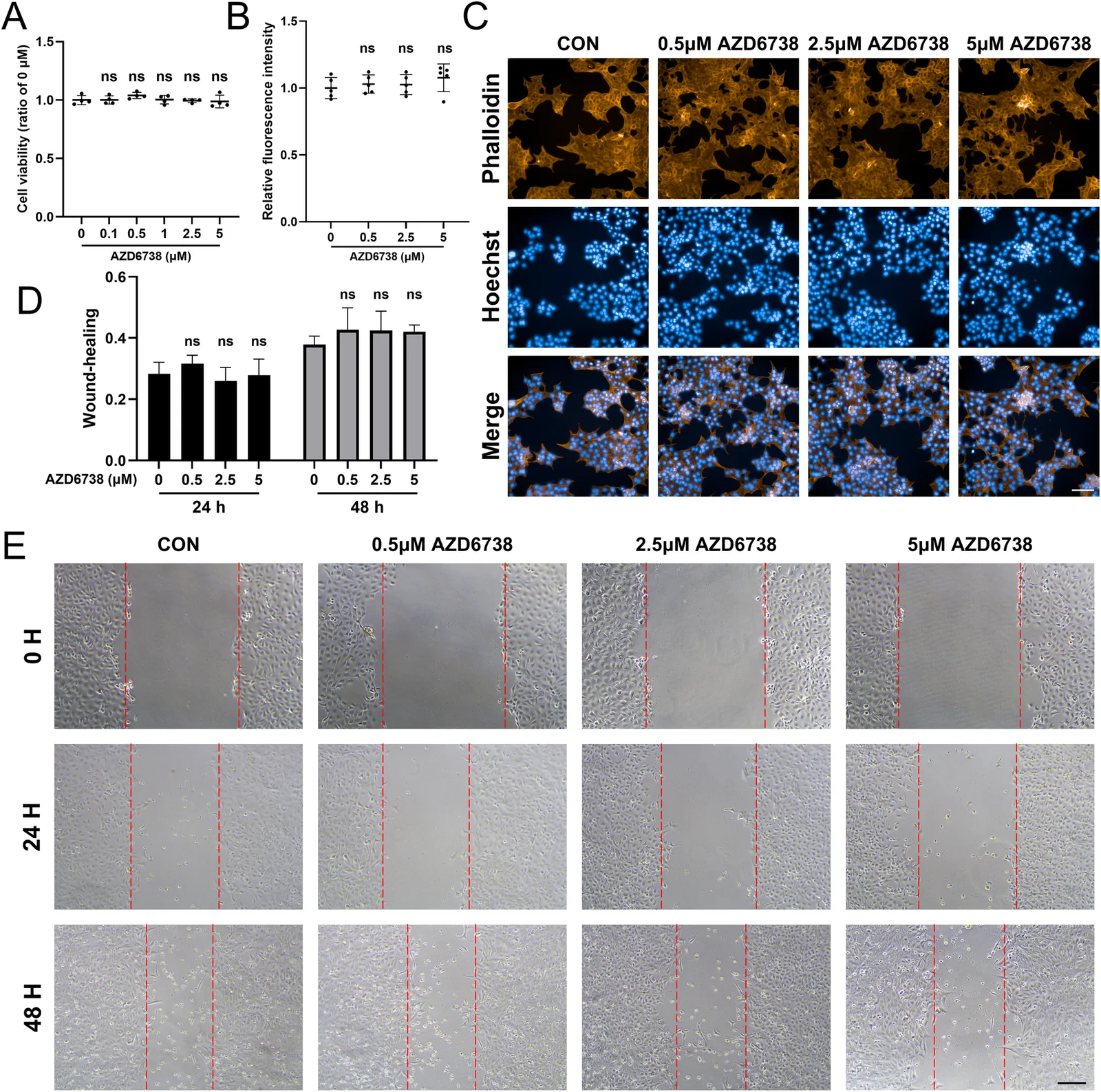
AZD6738 exhibits favorable biocompatibility with HCE-T corneal epithelial cells. **(A)** Cell viability of HCE-T cells treated with increasing concentrations of AZD6738 (0.5, 1, 2.5, 5 μM) for 24 h, assessed by CCK-8 assay. **(B)** Phalloidin staining (orange) detecting F-actin cytoskeletal morphology; DAPI (blue) marks nuclei. **(C)** Quantitative analysis of relative F-actin fluorescence intensity. **(D)** Quantitative analysis of wound healing rate. **(E)** Wound healing assay detecting HCE-T cell migratory capacity at 0, 24 and 48 h after AZD6738 treatment. Data are presented as mean ± SD (*n* ≥ 3). ns: no significant difference. Scale bars: 100 μm **(C)**; 200 μm **(E)**

Rhodamine-phalloidin staining showed no significant difference in F-actin distribution or morphology between any treatment group and the vehicle control, and relative fluorescence intensity did not differ significantly among groups (Figure 6B).

Scratch assay was performed to evaluate cell migration. Wound closure rates, calculated as (A_0h_ − A_48h_)/A_0h_, were recorded at 0, 24 and 48 h post-scratch; no statistically significant differences were observed between any AZD6738-treated group and the vehicle control at 48 h (Figures 6C,D). Within the concentration range and treatment time tested, no AZD6738 concentration differed significantly from the vehicle at 24 and 48 h.

## 4 Discussion

Phenotypic attenuation of LPS keratitis by AZD6738, including reduced corneal opacity, neovascularization and collagen deposition, was established in our companion study (28). In this study we characterized how AZD6738 reshapes the LPS-induced corneal transcriptome and identified the regulators that organize the response. LPS produced a transcriptomic shift dominated by gene upregulation, which AZD6738 substantially reversed: on average approximately 57% of LPS-induced changes were restored, consistently at the gene and pathway levels. The suppressed program was organized by inflammatory transcription factors, four of which (NFKB1, RELA, SPI1 and STAT1) were also significant in human bacterial keratitis. In RAW264.7 cells, p65 overexpression showed that AZD6738 reduces NF-κB-driven transcription and Ser536 phosphorylation.

The enrichment of innate immune and cytokine signaling in the LPS response is consistent with transcriptomic profiling of human infectious keratitis (32), in which innate immune programs likewise dominate the host response in human keratitis and persist into late-stage disease (17), and activation of resident innate immune cells is likewise recognized as a central mechanism in chronic ocular diseases such as glaucoma (33). Our analysis adds two elements to these descriptive profiles: the treatment effect is expressed on the same scale as the disease effect, and the response is resolved to the level of transcription factor regulons rather than only to affected pathways.

NF-κB has been a target of interest in bacterial keratitis, where inhibiting it reduces inflammatory infiltration and corneal damage (34), and NF-κB-dependent signaling also disrupts barrier function in cultured human corneal epithelial cells (35). Such approaches act on pattern recognition receptors or their immediate adaptors, whereas the effect described here acts at a kinase upstream of NF-κB phosphorylation. ATR-associated DNA damage response signaling promotes rather than restrains inflammatory activation (25, 36), so ATR inhibition would be expected to reduce inflammatory transcription, consistent with the effect observed here. The direction of this effect is nevertheless context-dependent. In DNA-damage-driven settings, ATR restrains replication stress and thereby limits the DNA damage that feeds inflammatory sensing, so that its inhibition amplifies interferon signaling (26, 27). In LPS-induced inflammation, the findings reported here instead indicate a signaling role for ATR downstream of TLR4, because ATR inhibition reduced NF-κB Ser536 phosphorylation in macrophages, a relationship also observed at the transcriptional level *in vivo*. The two contexts therefore engage different effector arms of ATR, and the direction observed here follows from the TLR4–ATR–NF-κB axis rather than from canonical DNA damage response function. Our data converge on Ser536 phosphorylation of p65, which was increased by p65 overexpression together with the downstream cytokine response and reduced by AZD6738. Phosphorylation at this residue has been linked to p65 activation (37) but can also be catalyzed by kinases other than IKK (38), so we interpret the reduction as an indication of decreased upstream signaling to p65 rather than as evidence that the residue mediates the transcriptional effect. The steps between ATR and the IKK complex remain unidentified.

Suppression was not confined to NF-κB, and JAK-STAT signaling was also enriched in the LPS response and negatively enriched after treatment (39, 40), suggesting that AZD6738 acts on a program-level regulatory state rather than on a single pathway. Reversal was asymmetric, with 51.2% of LPS-upregulated genes significantly reversed compared with 23.6% of LPS-downregulated genes, and no significant transcription factor was detected for the downregulated or treatment-upregulated directions. Expression of LPS-suppressed genes was therefore less readily restored, which may reflect the absence of an active driver for their re-expression within the 48 h window examined.

These results support ATR inhibition as a host-directed adjunct to antimicrobial therapy, an approach that does not depend on the antibiotic sensitivity of the organism and therefore provides a rationale in the context of resistance among corneal isolates (6). Delivery by subconjunctival injection with topical eye drops places the drug at the corneal surface (28, 41), and AZD6738 did not significantly reduce the viability or migration of human corneal epithelial cells over the concentration range tested. Because inflammation-induced damage to the corneal stroma, rather than the infection itself, leads to scarring and vision loss (5), an intervention that reduces the inflammatory transcriptional program while antibiotics control the organism addresses the component of the disease that antibiotics do not. The four conserved transcription factors are candidates for pharmacodynamic readouts in further development, although other host-directed targets are also under investigation (42, 43).

Several limitations qualify these findings. The design used three biological replicates per group at a single 48-h time point in an LPS-induced model, so dose- and time-dependence and the contribution of the additional virulence factors and neutrophil-driven responses present in live-bacterial keratitis were not captured; a 48 h regimen also does not reflect the days-to-weeks course of clinical keratitis. Bulk RNA sequencing cannot attribute expression changes to specific corneal cell types, and the cross-species validation relied on a small human dataset (four bacterial keratitis cases, seven controls) from FFPE tissue, establishing conservation of the regulator*y* axis rather than a therapeutic response. In addition, the *in vitro* experiments used a single AZD6738 concentration (2.5 μM), and neither viable cell counts nor per-cell readouts were acquired in parallel, so selective depletion of cytokine-active macrophages cannot be excluded as a contributor to the reduced cytokine mRNA levels. The model used intrastromal LPS rather than live pathogens, so the findings apply to endotoxin-driven inflammation and bacterial keratitis and do not establish efficacy against fungal or viral keratitis, although the ATR–NF-κB axis targeted here is engaged by multiple pathogen classes.

In summary, AZD6738 reverses a substantial part of the LPS-induced corneal inflammatory transcriptome, and the suppressed program is organized by inflammatory transcription factors whose regulons are conserved in human bacterial keratitis. The effect converges on NF-κB Ser536 phosphorylation, indicating decreased upstream signaling to p65 rather than establishing this residue as the mediator of the transcriptional effect. Because corneal scarring and vision loss in bacterial keratitis result predominantly from the host inflammatory response rather than from the organism itself, a pharmacological strategy that suppresses this response independently of antimicrobial susceptibility offers a rational adjunct in the setting of increasing drug resistance. The four conserved transcription factors identified here are candidates for pharmacodynamic readouts in further translational development.

## Data Availability

The datasets presented in this study can be found in online repositories. The names of the repository/repositories and accession number(s) can be found in the article/Supplementary Material.
