# Supplementary material for: AZD6738 reverses the LPS-induced corneal inflammatory transcriptional program through a conserved transcription factor network converging on NF-κB
Source: Front Med (Lausanne). 2026 Oct 26;13:2005671. doi: 10.3389/fmed.2026.2005671 (PMC13745219; doi:10.3389/fmed.2026.2005671)
Supplement: Supplementary file 1 [file Image1.pdf]

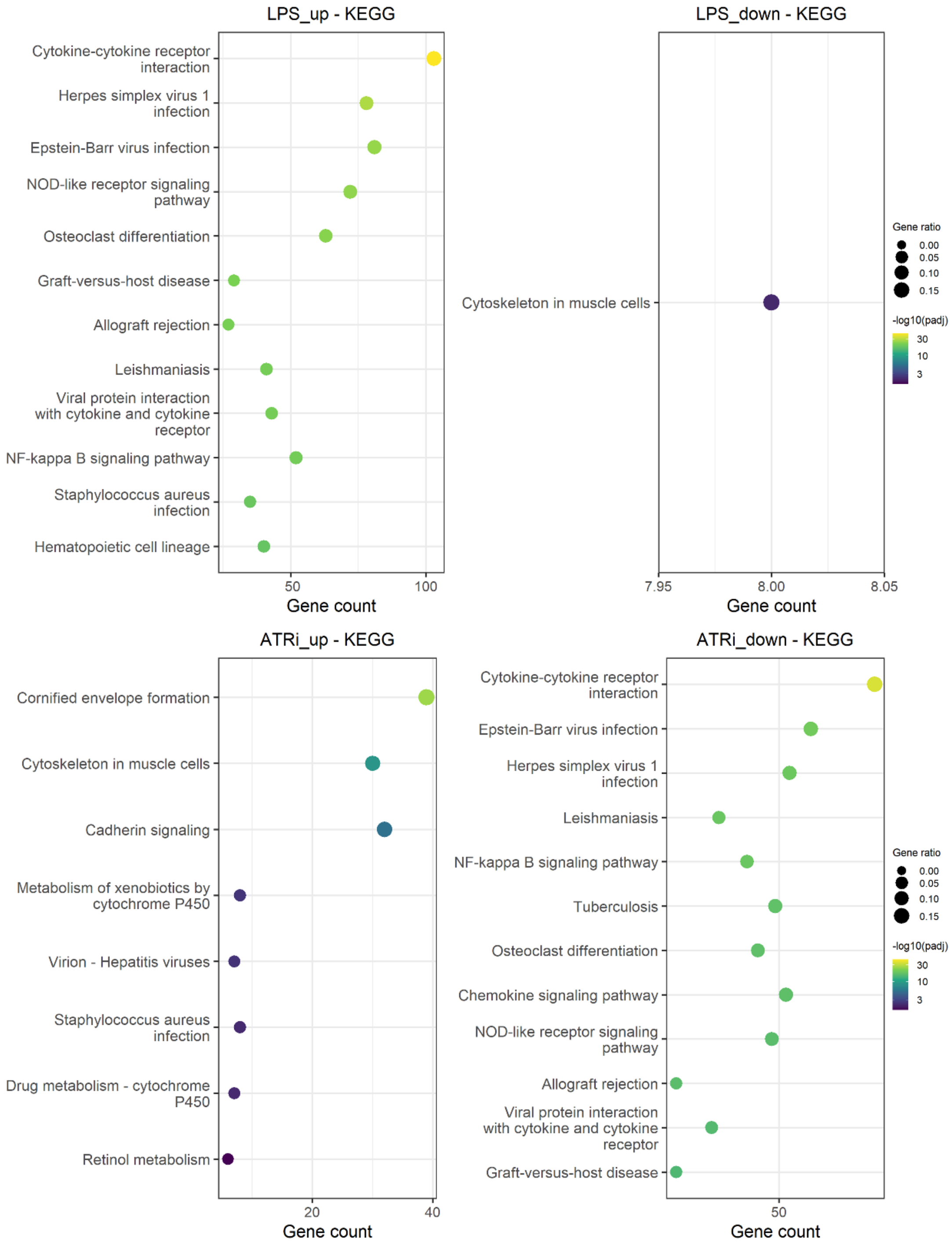

Supplementary Figure S1. KEGG enrichment analysis of the four differential gene sets. Top row, LPS effect (LPS_up, left; LPS_down, right); bottom row, treatment effect (ATRi_up, left; ATRi_down, right). All pathways shown are significantly enriched (padj < 0.05): 80 for LPS-upregulated, 1 for LPS-downregulated, 8 for treatment-upregulated, and 76 for treatment-downregulated genes; each panel shows the top 12 pathways by padj, or all pathways where fewer than 12 were significant. x-axis, gene count; bubble size, GeneRatio; colour, −log10(padj). The two panels of each row share one legend on the right.

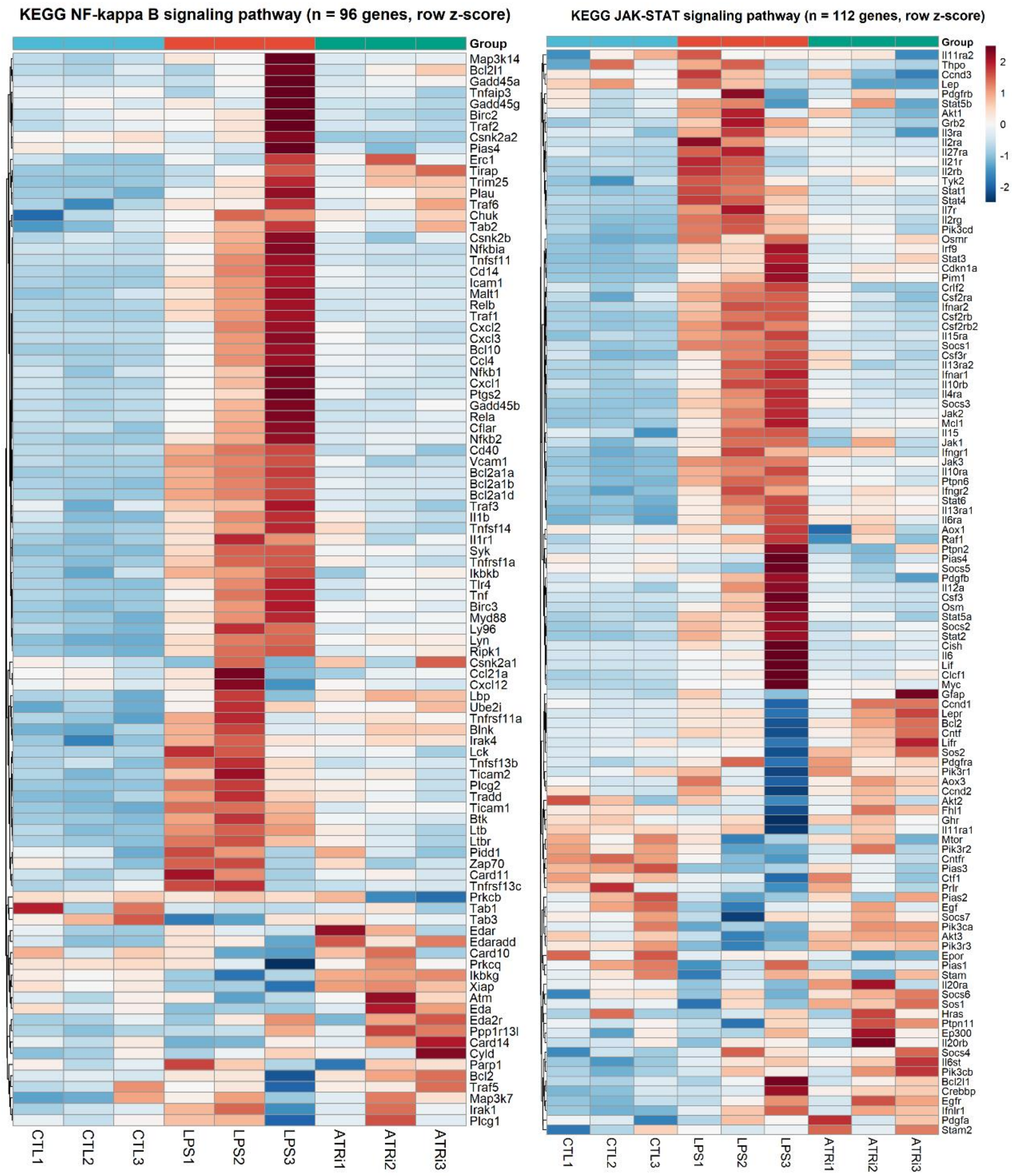

Supplementary Figure S2. Grouped expression heatmaps of KEGG pathway genes in the mouse corneal transcriptome. Left: NF-κB signaling pathway genes (n = 96); right: JAK-STAT signaling pathway genes (n = 112). Columns are arranged by group (CTL, LPS, ATRi).
