# Supplementary material for: AZD6738 reverses the LPS-induced corneal inflammatory transcriptional program through a conserved transcription factor network converging on NF-κB
Source: Front Med (Lausanne). 2026 Oct 26;13:2005671. doi: 10.3389/fmed.2026.2005671 (PMC13745219; doi:10.3389/fmed.2026.2005671)
Supplement: Supplementary file 2 [file Table1.pdf]

Supplementary Table S1. Primer sequences used for qRT-PCR

Gene	Forward primer (5′→3′)	Reverse primer (5′→3′)
Il6	TAGTCCTTCCTACCCCAATTTCC	TTGGTCCTTAGCCACTCCTTC
Tnf	CCCTCACACTCAGATCATCTTCT	GCTACGACGTGGGCTACAG
Il1b	GCAACTGTTCCTGAACTCAACT	ATCTTTTGGGGTCCGTCAACT
Gapdh	AGGTCGGTGTGAACGGATTTG	TGTAGACCATGTAGTTGAGGTCA
